# Video Games in ADHD and Non-ADHD Children: Modalities of Use and Association With ADHD Symptoms

**DOI:** 10.3389/fped.2021.632272

**Published:** 2021-03-12

**Authors:** Laura Masi, Pascale Abadie, Catherine Herba, Mutsuko Emond, Marie-Pier Gingras, Leila Ben Amor

**Affiliations:** ^1^Centre intégré universitaire de santé et de services sociaux du Nord-de-l'Île-de-Montréal (Hôpital-Rivière-Des-Prairies), Department of Psychiatry and Addictology of Université de Montréal (UdeM), Research Center of the Sainte-Justine University Hospital, Montreal, QC, Canada; ^2^Centre intégré universitaire de santé et de services sociaux du Nord-de-l'Île-de-Montréal (Hôpital-Rivière-Des-Prairies), Department of Psychiatry and Addictology of Université de Montréal (UdeM), Montreal, QC, Canada; ^3^Research Center of the Sainte-Justine University Hospital and of Université du Québec à Montréal (UQAM), Montreal, QC, Canada; ^4^Sainte-Justine University Hospital, Department of Psychiatry and Addictology of Université de Montréal (UdeM), Research Center of the Sainte-Justine University Hospital, Montreal, QC, Canada; ^5^Département de psychologie idem pour Catherine Herba, Université du Québec à Montréal (UQAM), Montreal, QC, Canada

**Keywords:** video game, ADHD, addiction, dependence, playtime, children

## Abstract

Video game addiction in young children is relevant, but it is especially important for children with ADHD. In order to obtain more data about the use of video games by Canadian children, and in particular by ADHD children, we explored the modalities of use (playtime, addiction score and usage by age) and compared them between ADHD and non-ADHD children. We then examined associations between addiction and ADHD symptoms and explored innovative results about the gender impact. Our study was cross-sectional, multicenter in child psychiatrist departments, exploratory and descriptive. We recruited three groups of children aged 4–12 years: the ADHD Group, the Clinical-Control Group and the Community-Control Group. For each group, the material used consisted of questionnaires completed by one of the parents. Data collection took place from December 2016 to August 2018 in Montreal (*n* = 280). Our study highlighted a vulnerability in ADHD children: they would exhibit more addictive behaviors with respect to video games (Addiction score: 1.1025 in ADHD Group vs. 0.6802 in Community-Control Group) and prolonged periods of use. We also observed a correlation between the severity of ADHD symptoms and excessive use of video games (*p* = 0.000). Children with severe ADHD showed significantly higher addiction scores and, in a multiple regression analysis a combination of gender and ADHD explained the excessive use of video games.

## Introduction

Children and adolescents report playing video games frequently, and we can see that this use is being reported at ever-younger ages (1). For example, 91% of children aged 2–17 are reported to play video games (2). New games on smartphones and tablets are rapidly being developed, many of which target young children including toddlers (3). The use of screens starts at an early age with more than 30% of children having used a tablet before the age of 2, and often for playing video games. From about the age of 4, the computer has become an increasingly popular medium for children to play video games (4). When children get older, a vast array of devices are used such as consoles, computers, tablets and smartphones, both online and offline.

The period between 4 and 12 years is therefore an important stage where children are increasingly exposed to video games and as such represents a relevant developmental period to study factors linked to excessive or addictive use of video games. Studies tend to show that ~2.0–5.5% of adolescents/young adults exhibit an addiction to video games (5). Multiple factors including the types of video games, personality characteristics and early exposure contribute to this addiction, but its origins are complex and gaps exist, particularly pertaining to such use by children (6).

In 2018, the World Health Organization (WHO) introduced Gaming disorder (GD) to the International Classification of Disease-11 (ICD-11) (7) and highlighted three symptoms: “impaired control over gaming, increasing priority given to gaming and continuation or escalation of gaming despite the occurrence of negative consequences” (8). Similarly, Internet gaming disorder (IGD) was included in the Diagnostic and Statistical Manual of Mental Disorders, Fifth Edition (DSM-5) in 2013, but it was categorized as requiring further study and not sufficiently well-established to be a part of the official classification of mental disorders for routine clinical use (9). IGD requires experiencing five or more of the following symptoms within a year: “preoccupation or obsession, withdrawal, tolerance, loss of control, loss of interest, continued overuse, deceiving, escape of negative feelings, functional impairment” (8). In this article, we will use the expression “video game addiction” which, in summary, shall be interpreted as a repeated use of video games that results in a significant impairment to an individual's social, family or professional life over a prolonged period of time.

The association between attention deficit hyperactivity disorder (ADHD) symptoms and video game addiction is observed among children and adolescents, but it is as yet not understood (10). The relationship between ADHD and the excessive use of video games may likely be bidirectional and needs to be clarified. In addition, most studies have been conducted among adolescents, and data for preschool children is almost non-existent apart from a 2018 study by Paulus et al. (11) ADHD is a risk factor for addiction in general (12), and it is the most frequent psychopathology in video game addiction (13). It is already listed in the DSM-5 as comorbid with Internet gaming disorder (9). However, the use of online games is mostly among adolescents and young adults and less in children who usually start with offline video games (4).

### Hypotheses and Objectives

We've seen the major role that video games play in today's society, particularly among teenagers but also among increasingly younger children. Video gaming is typically a leisure activity, but its practice among some people can turn into an addiction and have negative consequences. Following an overview of the literature, we observed that the majority of studies on this topic are cross-sectional and prospective. These studies are conducted among teenagers and young adults without a control group and have seldom targeted children and only very rarely young (preschool or early primary school-aged) children. The scales used to measure video game addiction are highly variable and unvalidated. Furthermore, diagnosis of ADHD or other comorbidities is not generally confirmed through use of validated scales or by a specialized doctor [(child) psychologist, pediatrician].

In this context and with a view to adding to knowledge about video gaming and its potential risks in potentially vulnerable populations, we sought to compare, in an exploratory manner, children aged 4–12 from a clinical population (divided into two subgroups, ADHD and non-ADHD) with children aged 4–12 from the general population through use of standardized, validated scales. Based on our hypotheses, we expected to see an increase in addictive video gaming behaviors and in duration of video game use among children in the ADHD subgroup in comparison to the community population. Another of our hypotheses was that the intensity of ADHD symptomatology correlates positively with addictive behaviors.

In order to better understand the relationship between video game use/addiction and ADHD in preschool/school children, we conducted a descriptive and exploratory study with the following objectives:

(1) To determine the modalities of use of video games (playtime, addiction score and usage by age) in children with ADHD compared to children without ADHD.
(2) To examine the associations between video game addiction and ADHD symptoms.
(3) To explore the gender difference in video game use, the type of video games played by children with ADHD and the impact of parents on gaming.

## Methods

### Participants

The participants were children aged 4–12 who comprised the three study groups. The first group was made up of children with ADHD (clinical group); the second was made up of children presenting one or more mental health diagnoses other than ADHD (clinical control group); and the third was made up of children from the general community (community control group). All participants' parents had to be able to speak and understand French. The only exclusion criteria were the inability to read, write and understand French, and the presentation of an intellectual handicap or active psychotic symptoms.

The age range of 4–12 was selected in order to more specifically target video gaming among school and preschool-aged children. It would be more difficult to direct our research questions at a broader age range in light of the inherent heterogeneous aspect already present in the children. With this in mind, the aspects evaluated as part of this study were not specific but rather related to individuals' general functioning.

Participants in the clinical and clinical control groups were recruited at the outpatient pediatric (ADHD and development) and child psychiatric (outpatient age 6–12, Tourette and age 0–5) clinics at the CHU Sainte-Justine, and the outpatient child psychiatric clinics at the Hôpital-Rivière-des-Prairies of the CIUSSS du Nord-de-l'Île-de-Montréal (CIUSSS NIM). These persons were recruited by a research assistant while waiting for an appointment in the waiting room at the outpatient pediatric or child psychiatric clinic. The assistant proposed participating in the study to all waiting patients and their parents and informed them about the project. The third group (community control group) was recruited at day camps in the Montreal, Laurentides and Lanaudière regions. Parents with their children were approached by a research assistant as they arrived at or left the day camp. The recruitment procedure followed was identical to that detailed above.

The exclusion criteria for parents and children were being unable to read, write and understand French. Participants ought not to have an intellectual disability or to be actively psychotic.

### Study Design

The study was cross-sectional, multicentre (CHU Sainte-Justine, CIUSSS du Nord-de-l'Île-de-Montréal) in child psychiatric departments, exploratory and descriptive. The material used consists of questionnaires to be completed by one of the parents for each group. Data was collected between December 2016 and August 2018 in Montreal. This study is a first step toward evaluating this type of problem. The tools used consisted of validated questionnaires (three questionnaires described below in the Measures section) to be completed by one of the parents in one sitting (with a 30-min estimated completion time). After obtaining consent, a numbered envelope was given to each participant containing the questionnaires and a recruitment letter). This letter restated the main information concerning the research project and the project procedure and provided the telephone number of the research coordinator at the CHU Sainte-Justine in the event that participants needed additional information.

In the clinical group, in addition to the questionnaires, participants' medical records were consulted once by a research assistant to verify psychiatric diagnoses. All ADHD and/or psychiatric diagnoses were made by a child psychiatrist from the CHU Sainte-Justine or the CIUSSS NIM. Diagnoses were arrived at following a comprehensive, multidisciplinary assessment based on the validated diagnostic criteria from the reference diagnostic guide in psychiatry in North America, the DSM-5 (Diagnostic and Statistical Manual of Mental Disorders).

### Measures

The tools used consisted of three questionnaires completed by one of the parents in one sitting: the Sociodemographic Questionnaire with added questions on video game use among children and parents, the SDQ (Strengths and Difficulties Questionnaire) and the Questionnaire sur l'attention et l'ordinateur (questionnaire on attention and computers) (QUATTORD).

#### Sociodemographic Questionnaire With Added Questions on Video Game Use

General sociodemographic data were documented, including participant age, sex, current education level or last level achieved, family status, ethnic origin, etc., as well as the time of year of questionnaire completion. A standard sociodemographic questionnaire was used to collect this information supplemented by questions about screen time and, in particular, video game use. The added questions included questions about total screen time devoted to video gaming or to watching movies or television during the week and on weekends/holidays, frequency of use by game type and number of electronic devices in the child's possession. We added questions concerning video game use that we then categorized based on gaming habits. Although there was no consensus concerning this classification, video games can generally be divided in the following groups: creative games, educational games, non-violent character-based games and violent character-based or non-character-based games. We also added questions to this questionnaire for parents about their own video gaming habits and their perceptions concerning their children's gaming habits with a view to collecting preliminary data to guide our subsequent research questions (14).

### Psychometric Data

#### QUATTORD (Questionnaire de l'attention et de l'ordinateur)

This is a questionnaire to evaluate ADHD symptoms and video game use. It includes 19 questions on ADHD symptoms (a 4-point Likert scale on which 0 = not at all and 3 = very much), nine questions on screen use (descriptive) and 11 questions on video game addiction symptoms (4-point Likert scale on which 0 = never and 3 = always). The questions on ADHD symptoms and addiction symptoms are based on the diagnostic criteria from the DSM-5 (9), and this questionnaire is easy to complete. It was used by Paulus in a study on ADHD and video gaming among more than 1,000 school and preschool-aged children and has been used since the launch of that multinational study (15). The results obtained using this questionnaire were used to generate empirical data aligned to the data in the literature.

#### SDQ (Strengths and Difficulties Questionnaire)

The SDQ is a validated questionnaire consisting of 25 items evaluating strengths (e.g., prosocial behaviors) and difficulties (e.g., hyperactivity, emotional regulation difficulties) in children aged 4–16 (16). We used the version to be completed by parents (there is also a version for teenagers and one to be completed by teachers). The 25 items are divided into five categories (emotional symptoms, conduct problems, attention deficit/hyperactivity, peer problems, and prosocial behaviors), each with five questions. The original, validated English version of this instrument has sound psychometric properties, which are found in the French version. Cronbach's alpha for the total difficulties score was acceptable at 0.77 (17). The goal was to use an additional independent assessment (independent from the QUATTORD) to obtain an overview of the children's general internalizing and externalizing behaviors.

### Ethical Approvals

The research project was approved by the research ethics board of the CHU Sainte-Justine (the main project center). Confidentiality and anonymity were maintained throughout the study and during data transcription. The participants' identities were coded using the numbers indicated on the envelopes. The questionnaire results were organized in an Excel spreadsheet and identified using these numbers. The computer holding this data is stored under lock and key in the research offices of the recruitment centers. If parents agreed voluntarily to participate after explanations were provided, they could then sign the consent form. Consent forms were signed in duplicate with one copy given to the parents. We also asked the children for their consent.

### Statistical Analysis Strategy

First, ANOVA (analysis of variance) were conducted to compare video game playtime and addiction scores according to QUATTORD between the three study groups and between age categories for each group (4–6, 6–8, 8–10, and 10–12 years). We then clarified the differences identified by *post-hoc* analyses (Hochberg and Games-Howell). Correlation analyses were then used to assess associations between the severity of ADHD symptoms and SDQ items in relation to dependency. We then conducted a multiple regression analysis to explore the combination between the gender and having ADHD or a clinical diagnosis.

Lastly, we used multiple linear regression to evaluate associations between playtime and addiction score (continuous dependent variables obtained *via* the QUATTORD) and income, parental video gaming habits and parental perceptions concerning the impact of video gaming on their children (categorical independent variables obtained *via* the sociodemographic questionnaire).

The statistical significance threshold was α = 0.05.

## Results

### Descriptive Analyses

A total of 280 participants completed the study: 98 participants (35.0%) in the ADHD Group, 37 participants (13.2%) in the Clinical-Control Group and 145 in the Community-Control Group (51.8%) (Table 1). Participants were exclusively French-Canadian Caucasians. The mean age was 7.68 years and there was no significant difference between the three groups with respect to age (*p* > 0.005). With regard to gender, the proportion of boys and girls in the study for each group is also presented in Table 1.

**Table 1:** Proportion of boys and girl in each group.

**Gender**	**Total**	**ADHD group**	**Clinic-control group**	**Community-control group**
Boys	183 (65.4%)	79 (80.6%)	31 (83.8%)	73 (50.3%)
Girls	97 (34.6%)	19 (19.4%)	6 (16.2%)	72 (49.7%)
Total	280	98 (35.0%)	37 (13.2%)	145 (51.8%)

### Modalities of Use Between ADHD and Non-ADHD Children

ANOVA showed that the video game playtime was significantly higher for the ADHD Group compared to the Community-Control Group during both weekdays and weekends. The Hochberg and Games-Howell *post-hoc* analysis between the three groups were significant and identical with regard to video game playtime on weekdays and weekends. They indicated a longer time spent on video games in the ADHD Group (*p* = 0.002 for ANOVA and *post-hoc* analysis) (Table 2).

**Table 2:** Playtime during the week and the weekend.

**Group**	**Mean number of hours during the week**	**Mean number of hours during the weekend**	***p*-value with respect to Community-Control Group**
ADHD group	2.05	3.01	0.002 for week and weekend
Clinical-control group	1.86	2.93	0.335 for week and 0.135 for weekend
Community-control group	1.44	2.36	NA

*Statistically significant p ≤ 0.05*.

We compared video game addiction scores obtained at the QUATTORD by ANOVA. Addiction was significantly higher for the ADHD Group compared to the Community-Control Group (*p* = 0.000 for ANOVA and *post-hoc* analysis) (Table 3).

**Table 3:** Addiction scores for video games.

**Group**	**Addiction score**	***p*-value with respect to community-control group**
ADHD group	1.1025	0.000
Clinical-control group	0.9355	0.119
Community-control group	0.6802	NA

*Statistically significant p ≤ 0.05*.

For all groups, we compared the time spent on video games between age categories by ANOVA. Since differences were only significant in the ADHD Group, we then conducted a *post-hoc* analysis in this group only. We found that time spent on video games was significantly higher for the 10–12 age group compared to the 4–6 age group during weekdays and weekends (Table 4).

**Table 4:** Usage during the week and the weekend among age categories of the ADHD group.

**Age category**	**Mean number of hours during the week**	**Mean number of hours during the weekend**	***p*-value with respect to the 10–12 years-old age category**
4–6 years-old	1.54	2.32	0.005 for the week and 0.000 for the weekend
7–9 years-old	1.95	2.95	0.027
10–12 years-old	2.69	3.79	NA

*Statistically significant p ≤ 0.05*.

### Associations Between the Severity of ADHD Symptoms and Video Game Addiction

There was a significant correlation (Pearson correlation) for each ADHD symptom (impulsivity, inattention, hyperactivity) and video game addiction. The strongest correlation was with impulsivity (Table 5).

**Table 5:** Correlation between symptoms of inattention, hyperactivity, impulsivity, and video game addiction.

**Symptoms**	**Video game addiction**	***p*-value**
Inattention	0.279	0.000
Hyperactivity	0.294	0.000
Impulsivity	0.310	0.000

*Statistically significant p ≤ 0.05*.

There was a significant correlation (*r* = 0.182 for *p*-value = 0.003) between hyperactivity symptoms and weekend use time. There was no significant correlation for the other symptoms (inattention and impulsivity) and the time of use during the weekend. Similarly, we did not find a positive correlation for ADHD symptoms and the time of use of video games during the week.

### Relationship Between SDQ Items and the Use of Video Games

All correlations between SDQ items and video game addiction scores found by the QUATTORD are highly significant with *p* < 0.01: positive correlations with emotional problems (0.321), conduct disorders (0.293), hyperactivity (0.242) and peer problems (0.201); and negative correlation with prosocial abilities (−0.272).

Correlations between SDQ items and playtime during the week are positive and significant for hyperactivity (0.151) and peer problems (0.143) as well as emotional problems (0.208, highly significant for this last correlation with *p* < 0.01). Correlations between SDQ items and playtime during the weekend are positive and highly significant (*p* < 0.01) for emotional problems (0.242), conduct disorders (0.164), and hyperactivity (0.229).

### Exploratory Results

According to our regression analyses where we looked for variables predicting addiction, we found a significant interaction between addiction, clinical groups and gender, based on a linear regression (0.533). That is, the boys in the study showed more dependence if they belonged to one of the two clinical groups (ADHD Group and Clinical-Control Group) and, more specifically, ADHD boys had the highest addiction scores. We did not find this trend for girls with equivalent addiction scores, regardless of the group (Clinical-Control Group or Community-Control Group).

With respect to the types of games played, 62 participants (24.8%) often played creative games (e.g., Minecraft), 66 participants (26.5%) often played educational games (e.g., Oregon Trail), and 51 participants (8.4%) often played violent games (e.g., Call of Duty) (Table 6).

**Table 6:** Types of video games played most frequently.

**Game type**	**Frequency played**			
	**Never**	**Rarely**	**Sometimes**	**Often**
Creative	19.2%	29.2%	26.8%	24.8%
Educational/concentration	14.9%	21.3%	37.3%	26.5
Violent human-like	62.9%	13.1%	15.5%	8.4%
Violent cartoon-based	51.2%	19.4%	19.4%	17.5%
Non-violent character-based	22.9%	17.3%	35.3%	24.5%

With respect to parents, we found that only 17.4% of parents played video games. A negative association was then identified between average household income with addiction score and children's playtime, indicating that video game use (playtime and addiction) varies inversely with income. This corresponds to the data from the scientific literature indicating that a low socioeconomic stratum is associated with development of video gaming addiction and increased playtime (18). Using linear regression, we then identified positive correlations between video game use among children (playtime and addiction score) and parental perception of the negative impact of video gaming on their children (excessive playtime and conduct problems after playing video games) (Table 7).

**Table 7:** Multivariate linear regression of income and parental perception with children's playtime and addiction score.

	**Playtime during week (hours)**		**Playtime on weekends (hours)**		**Addiction score**	
	**B**	**p**	**B**	**P**	**B**	**p**
Household income (1–6)	−11.9	0.000	−17.3	0.002	−0.9	0.051
Do you find that your child spends too much time playing video games? (No = 0; Yes = 1)	28.3	0.008	53.4	0.000	4.5	0.000
Do you find that this has positive or negative impact on your child's behavior? (Neutral/negative = 0; Positive = 1)	38	0.009	50.4	0.003	2.8	0.019

## Discussion

Research on the relationship between ADHD and video game addiction has mainly been conducted among adolescents and young adults, and studies like ours that focus on children are rare (19). Our study highlighted a vulnerability in ADHD children as they exhibit more addictive behaviors with respect to video games and demonstrate prolonged periods of use. We also observed a correlation between the severity of ADHD symptoms and excessive use of video games. Finally, our results suggest that the association between males and ADHD is an additional risk factor for the excessive use of video games.

### Modalities of Use and Comparison Between ADHD and Non-ADHD Children

The duration of use was significantly higher in the ADHD Group compared to the Community-Control Group, during both the week and the weekend. We also found that, in accordance with the literature, when ADHD symptoms are more severe, playtime would be significantly longer (20). These results indicate that caution must be exercised when children with ADHD play video games.

Furthermore, the degree of video game addiction was also significantly higher in children with ADHD compared to children in the community group. In the former, we indeed found, as early as preschool age, a higher tendency to develop addictive behaviors toward video games compared to children of the same age without ADHD. This would be even higher in ADHD children with behavioral problems since they might be less able to accept a certain control over their playtime. In addition, ADHD patients are at risk of addictive behavior and, more specifically, an ADHD diagnosis should increase the risk of being dependent on online video games (21).

The explanatory mechanisms of this attraction by ADHD patients to video games include becoming bored quickly, intolerance to waiting, difficulties with self-control, difficulties being motivated, the need for intense stimulation and difficulties in interpersonal relationships (22). On the other hand, studies in neurobiology have shown a release of striatal dopamine involving the brain reward circuits during video game use improving the ability to concentrate during playtime which would provide a sense of comfort for young people with ADHD (23, 24). Risk factors for the development of addiction to video games are also typical traits of ADHD (impulsivity, difficulty in managing emotions and lack of prosocial capacity, for example) (25). Video games also seem to allow young ADHD patients to offset the frustrations and failures of real life with the successes and achievements they perceive while playing, which largely explains their appeal (22).

### Comparison of Usage by Age

The only significant difference found was in the ADHD Group for the 10–12 age category compared to the 4–6 age category with a longer playtime among 10–12 year-olds during the week and the weekend. According to Lemmens, the younger the video games are played, the higher the risk of developing addiction during adolescence, a period of vulnerability to addictive behavior (26). Indeed, early and regular exposure to video games with long playing sessions is one of the most common risk factors for cyberaddiction (27). According to our results, this risk factor is even more present in the ADHD Group, which presents at its core vulnerabilities to addiction.

ADHD children seem to show an increase in video game playtime as they get older, while the other two groups of children do not differ by age. This may mean that early exposure by a young ADHD patient is a risk factor for increasing use as they get older, especially at the onset of puberty and during adolescence. Indeed, adolescence is a high-risk period for addictions among young ADHD patients (12).

### Associations Between ADHD Symptoms and Video Game Addiction

We observed a significant correlation between all ADHD symptoms and those of video game addiction. According to Yen, ADHD symptoms (inattention, hyperactivity and impulsivity) among ADHD patients who are also cyberaddicts would indeed appear with more intensity than in ADHD patients who are not. More specifically, inattention seems to be the most aggravated symptom of video game abuse (28). However, in our study, impulsivity appears to be the most correlated to video game addiction. This is consistent with Bioulac's hypothesis that ADHD children would also have more behavioral problems with impulsivity related to video games (29). In the same vein and with regard to the impact of screen use, the more often young people consult their smartphone, the greater the risk of developing ADHD symptoms (30).

Thus, there appears to be a link between ADHD symptoms and the excessive use of video games, although at this stage it is not possible to predict the direction of causation. Moreover, it is important to differentiate the ADHD symptoms that result from an excessive use of video games from ADHD as a neurodevelopmental disorder.

### Association Between SDQ Items and the Use of Video Games

We found a highly significant positive association between behavioral problems on one hand, and video game addiction and playtime during the weekend on the other. The uncontrolled use of screens during childhood, whether for video gaming or otherwise, would therefore lead to a high risk of behavioral difficulties. Similarly, according to a study by Poulain et al. among children aged 2–6, the use of screens (including video games) is a major risk factor for the development of behavioral difficulties (31). Moreover, there is a strong association between early exposure to screens and the subsequent development of aggressive and antisocial behaviors (32, 33). In light of all those results and ours, it appears that excessive use of video games may negatively influence emotional and behavioral problems and the well-being of children from their early years.

We obtained a positive and significant correlation between socialization difficulties and video game addiction and playtime during the week, while the association between video game addiction and prosocial abilities was negative. Indeed, according to the literature, there is a strong association between increased screen time and reduced social development in children (34). Socialization difficulties are risk factors for video game addiction (27) and at the same time, they will be encouraged by the use of screens by pushing young people to overuse them in avoidance behaviors. Among young people who are not socially at ease and experience a sense of failure in their lives, online interactions reduce negative feelings such as loneliness and boredom (6). That being said, when parents limit and monitor screen time, children would develop better prosocial skills.

### Exploratory Results

#### Genre

We looked for the implication of gender and more specification for the use of video games. First, a majority of boys were in both clinical groups (ADHD Group and Clinical-Control Group). This corresponds to the clinical and epidemiological reality where the gender ratio for ADHD is two boys for one girl and where other neurodevelopmental disorders are also predominantly found in males. Moreover, according to Paulus et al., more boys than girls have video game consoles, and more girls than boys are non-players (11). We looked for covariables that influence addiction through a regression analysis. According to this regression, if we look at the interactions between groups and gender, ADHD boys appear to be the most at risk of video game addiction compared to boys in the other two groups. On the other hand, there does not seem to be any differences with respect to girls. Boys with a child psychiatry diagnosis of ADHD, therefore, seem to be the most vulnerable to video game addiction. It would then be a question of orienting the evaluation and care according to this fragility, which should not be neglected.

#### Video Games

It should be noted that studies on video games do not usually take into account the type of video games played, yet it seems essential to begin to make such distinctions if we want to make accurate recommendations. There are indeed many different types of video games and different ways of playing that likely have an impact on potential overuse. In this preliminary study, we tried to describe the games played by the children in order to get an initial overview of the situation. We found that the most popular video games played among 4–12 year-olds were educational rather than violent games. Educational games (those designed for a primary purpose other than pure entertainment) have pedagogical virtues that can be particularly useful for children with learning difficulties (35). This type of data can be used to refine future research and analysis in order not only to prevent the negative aspects of video games, but also to optimize their use.

The data from the literature also suggest that the relationship between ADHD symptom severity and video game addiction severity depends on the type of game preferred or played most often. This relationship may also depend on the level of reinforcement of the most-played game; that said, the reinforcement strategies of video games are constantly evolving to attract more and more players and become as addictive as possible. These changes have been bolstered by the initial success of massively multiplayer online role-playing games (MMORPGs), which have strong reinforcement structures, followed by other games that have increased reinforcement levels while creating a potentially higher risk of developing excessive use (e.g., Fortnite).

#### Parental Impact

No data exists regarding the use of video games by parents. In our study, we found that a majority of parents do not play video games at all. This has an impact on the parents' understanding and management of video games. Ideally, it is recommended that screen time be shared between parents and children according to the Canadian Pediatric Society guidelines (36). Poor relationships with parents, poor parental control, hostile parenting, and lack of rules on screen use are risk factors for video game addition. Parents must serve as role models with regard to screen use (37).

We identified a negative association between income with addiction score and playtime, indicating that video game use (playtime and addiction) varies inversely with income. This corresponds to the data from the scientific literature indicating that a low socioeconomic stratum is associated with development of video gaming addiction and increased playtime (38). Radesky and Christakis hypothesizes that parents from lower socioeconomic strata have fewer educational requirements in relation to video game use. Additionally, these parents appear to use video games as a pastime more frequently (39). Our results reflected this observation.

We explored video game use by parents, since the literature was weak in this area (37). Parental modeling and educational guidelines for video gaming appear to have an impact on IGD. The Canadian Pediatric Society (CPS) also recommends that parents use screen time to create opportunities for shared activities with children, and a majority of parents in our study did not play video games (37). We also identified a positive correlation between video gaming among children (playtime and addiction score) and parental perception of excessive playtime among their children. When it comes to estimating their children's playtime, parents appear to tend to overestimate the time their children dedicate to socially desirable activities (e.g., reading, homework) and underestimate the time they spend on socially undesirable activities (e.g., television, video games) (40). Moreover, many parents find that the official recommendations concerning children's screen time from pediatric learned societies such as the American Academy of Pediatrics (AAP) or the CPS are not realistic and could never be put into practice (41). They assert that they do not know how much screen time or, in particular, video game playtime is healthy or excessive (42). Parental evaluation of screen time in our study questionnaires may be erroneous if parents are confused about the recommendations or biased by social desirability.

We found a positive correlation between video game use in children (playtime and addiction score) and parental perception of negative impact on their children's behavior after playing video games. To our knowledge, the links between video game use among children and this negative perception have never been discussed. As part of this exploratory analysis, we were consequently able to collect preliminary data on this topic, and it may be interesting to look at other possible correlations based on the data collected through our questionnaires. It is important to note that these results apply only to a clinical population (clinical and community groups). Although parental concerns are underscored, they may also be somewhat directed in that parents completing the scales for the project could be particularly concerned about this issue in relation to their children. According to Schneider et al., a positive perception of video gaming could lead to more frequent usage problems in children (43), whereas others have indicated that a positive perception does not have any impact. Regardless, the concept of perception remains highly subjective, multifaceted and difficult to capture.

### Time of Year of Questionnaire Completion

We wanted to include this information, as it could create a bias in relation to data collection concerning video game use in that the majority of questionnaires were completed during the summer, when, based on our observations in the clinical setting, in the context of vacation and the absence of a structured schedule, children very likely spend more time playing video games. Recruitment levels were also higher due to the availability of research students.

## Strengths and Limitations

### Strengths

Although certain limitations and strengths were identified, we wish to take the reflection process further. The study has a certain number of strong points, such as the facts that the population was made up of children at least 4 years of age in the literature on video gaming and that the study was multicentre to ensure that the population was as varied as possible, thereby corresponding more closely to the general population. Our study is also very interesting in that it included control groups, the use of which enhances validity, particularly since the study involved evaluating behaviors in a specific segment of the population. The comparisons in the study are all the more relevant and useful since, in addition to the community group, we had a clinical control group, making even more detailed comparisons possible. From a statistical viewpoint, we took care to perform, with support from statisticians, appropriate analyses for our variables taking our hypotheses into account. We were successful in fully characterizing the population and the data on parents. Lastly, we were able to establish a highly comprehensive overview of the current knowledge based on a review of the literature.

We used multiple validated, reliable, standardized measures; a wide variety of measurement scales on video game addiction have been used in research on that topic, but none has been validated. Additionally, numerous studies have used non-representative, self-selected samples and/or small samples. This both compromises the comparability of results and raises questions as to diagnosis validity in participants. In this regard, the lack of consensus and use of overly broad criteria imply the overly broad use of the diagnosis of video game addiction in cases that could instead be classified as excessive behaviors not resulting in functional impairment and falling within the societal reality of screen use.

Similarly, in contrast to the majority of ADHD studies, in which the diagnosis is not confirmed, the ADHD diagnosis of our participants was made following comprehensive assessment at specialized clinics. The same applies to diagnosis in the clinical control group. What is more, the use of multiple comprehensive questionnaires further supports the validity of our results.

### Limitations

The results of this study should be interpreted in light of its limitations. Since the questionnaires are completed by the parents themselves, there may be a measurement bias with regard to their subjectivity. In addition, the possibility of an ADHD diagnosis in the community population has not been eliminated and could lead to a bias in the results. Lastly, it must be noted that the study is not longitudinal, and we were also not able to test any mechanisms that could explain patterns of findings. Additionally, questions are asked only about video game use at T0 of questionnaire completion, which does not allow for a broader perspective over time. Playtime may also be underestimated, since the measures are based on scales completed by the parents, who may have been influenced by lack of knowledge about actual playtime and/or social desirability to indicate values lower than actual. The possibility of ADHD diagnosis in the community population was not ruled out and could lead to a bias in the results.

### Perspectives and Reflection

The data collected on video gaming and parents are exploratory. These topics are of interest, and based on these initial results, we can now delve further into our hypotheses and direct our research questions. Next, diagnoses in the clinical control group were documented but not analyzed and could be incorporated into future subanalysis efforts to explore the impact of other diagnoses in child psychiatry (also by expanding recruitment in this subgroup). In addition, the questionnaires were completed by only one of two parents, which could lead to different responses if parents were separated or had different approaches to managing screen time (which we see frequently in the clinical setting). However, in our characterization of the population, we noted that a large majority of parents in our sample were not separated. We also did not track refusals, and the parents who consented appeared very willing to assist, which could indicate underlying problems (at least in their view).

This project lacks a longitudinal aspect, which is conspicuously absent from the literature. To create the most accurate portrait possible of video game use, this activity would have to be tracked over time, while also avoiding situational use. For example, we observed that the majority of recruitment took place in the summer, whereas use doubtless varies depending on whether school is in session or children have a structured schedule as well as their options for outdoor activities.

The current COVID-19 pandemic has greatly affected lifestyles and screen time. Already omnipresent in our lives, screens have become the only means of working, entertaining ourselves, keeping in touch with loved ones, socializing and learning. Moreover, screens became a go-to solution for keeping children busy while parents continued working from home and daycare facilities and schools remained closed. With regard specifically to video gaming during the pandemic, video game use increased significantly during lockdown periods (also generating exponential earnings growth over a period of a few months for the video game industry), but this greater use could also be only temporary.

A next step would be to undertake analyses and explore the impact of video games on family dynamics and academic performance, document the types of video games used and identify any comorbidities potentially influencing this use. Then, although we have studied the adverse effects of video gaming in terms of addiction, it would be useful nonetheless to examine any benefits it may also have. For example, video games may also help to develop certain skills (sense of control, socialization, coordination, etc.) (44) and offer new educational and therapeutic possibilities.

Knowledge about video game addiction continues to grow, and we continue to learn more about potential vulnerability to this addiction among certain individuals based on their mental health diagnosis. In particular, an ADHD diagnosis appears to be a major factor in the development of a video gaming addiction, although this association also appears bidirectional and is not yet fully understood. At the same time, studies on the relationship between ADHD and video game addiction have been conducted primarily in teenagers and young adults and have rarely targeted children. Our study consequently underscores the vulnerability of children with ADHD to excessive video gaming and the consequences of gaming on their symptomatology. We wanted to supplement the analysis component of our study by exploring the role of internal (age, sex) and external (socioeconomic stratum, parents' role) factors, thereby corroborating the concept of a multifaceted relationship behind addictive video gaming behaviors.

## Conclusion

ADHD symptoms and video game addiction appear to have a bidirectional relationship in which the ADHD symptoms make video gaming appealing, while play itself exacerbates the ADHD symptoms by providing an activity that continually reinforces the need for instant gratification. Long hours of video gaming may further reinforce and consolidate children's propensity to uncontrolled reactivity and pervasive impatience without coaching to develop more reflection-oriented behaviors. As stated previously, the time dedicated to video gaming is spent at the expense of leisure activities such as sports, music and the arts, which would assist in developing children's attention, self-assurance, behavioral inhibition, discipline, team skills, and socialization. In this context, related studies provide opportunities to reflect on the potential impact on our practice. By exploring the consequences of intensive video gaming for these young children, it is possible to develop intervention approaches to propose to professionals.

Although we understand that video games present risks, it would still be interesting to look at the benefits they can bring. Indeed, it is important to note that they can also help in developing skills such as a sense of control and coordination (45). There is a definite interest in using them as a lever for young people by offering new educational and therapeutic perspectives.

## Data Availability Statement

The raw data supporting the conclusions of this article will be made available by the authors, without undue reservation.

## Ethics Statement

The studies involving human participants were reviewed and approved by Centre de recherche du CHU Sainte-Justine. Written informed consent to participate in this study was provided by the participants' legal guardian/next of kin.

## Author Contributions

All authors listed have made a substantial, direct and intellectual contribution to the work, and approved it for publication.

## Conflict of Interest

The authors declare that the research was conducted in the absence of any commercial or financial relationships that could be construed as a potential conflict of interest.

## References

[1] Zero to Eight. Children's Media Use in America. Rideout, V. A Common Sense Research Study. (2017) Available online at: https://www.commonsensemedia.org/research/the-common-sense-census-media-use-by-kids-age-zero-to-eight-2017 (accessed July 21, 2020).

[2] The NPD Group. The Video Game Industry Is Adding 2–17 Year-Old Gamers at a Rate Higher Than That Age Group's Population Growth. Available online at: http://www.afjv.com/news/233_kids-and-gaming-2011.htm (accessed April 28, 2019).

[3] Courage ML Troseth GL. Infants, toddlers and learning from screen media. In: Tremblay RE Boivin M Peters RDeV Rvachew S, editors. Encyclopedia on Early Childhood Development. Available online at: http://www.child-encyclopedia.com/technology-early-childhood-education/according-experts/infants-toddlers-and-learning-screen-media. Published November 2016 (accessed September 5, 2019).

[4] Duggan M Brenner J. The demographics of social media users. Pew Internet Project. (2013) 2013:1–4. Available online at: http://pewinternet.org/Reports/2013/Social-media-users.aspx

[5] Paulus FW. Internet gaming disorder in children and adolescents: a systematic review. Dev Med Child Neurol. (2018) 60:645–59. 29633243 10.1111/dmcn.13754

[6] Rossé E. Les joueurs problématiques de jeux vidéo: éléments cliniques. In: Venisse JL Bronnec MG, editors. Les addictions sans drogue: prévenir et traiter, 16. Nantes: Elsevier Masson (2012). p. 127–32. 10.1016/B978-2-294-71136-7.00016-7

[7] World Health Organization. International Classification of Diseases 11th Revision (ICD-11). Geneva: World Health Organization (2018).

[8] Jo YS Bhang SY Choi JS Lee HK Lee SY Kweon Y-S. Clinical characteristics of diagnosis for internet gaming disorder: comparison of DSM-5 IGD and ICD-11 GD diagnosis. J Clin Med. (2019) 8:945. 10.3390/jcm8070945 31261841 PMC6678371

[9] American Psychiatric Association. Diagnostic and Statistical Manual of Mental Disorders. 5th ed. Washington, DC: American Psychiatric Publishing (2013). 10.1176/appi.books.9780890425596

[10] Wan CS Chiou WB. Why are adolescents addicted to online gaming? An interview study in Taiwan. Cyberpsychol Behav. (2006) 9:762–6. 10.1089/cpb.2006.9.762 17201603

[11] Paulus FW Sinzig J Mayer H Weber M von Gontard A. Computer gaming disorder and ADHD in young children—a population-based study. Int J Mental Health Addiction. (2018) 16:1193–207. 10.1007/s11469-017-9841-0

[12] Ginsberg Y. The unmet needs of all adults with ADHD are not the same: a focus on Europe. Expert Rev Neurotherapeutics. (2014) 14:799–812. 10.1586/14737175.2014.926220 24894408

[13] Han DH Lee YS Na C Ahn JY Chung US Daniels MA. The effect of methylphenidate on Internet video game play in children with attention-deficit/hyperactivity disorder. Comprehensive Psychiatry. (2009) 50:251–6. 10.1016/j.comppsych.2008.08.011 19374970

[14] Masi L Herba C Garel P. Projet pilote: exploration de l'utilisation d'Internet et des medias sociaux chez un groupe d'adolescents ayant participé a‘Espace Transition. Ann Medico Psychologiques. (2019) 177:319–26. 10.1016/j.amp.2018.04.009

[15] Paulus FW Ohmann S Von Gontard A Popow C. Internet gaming disorder in children and adolescents: a systematic review. Dev. Med. Child Neurol. (2018) 60:645–59. 10.1111/dmcn.13754 29633243

[16] Goodman R. The strengths and difficulties questionnaire: a research note. J Child Psychol Psychiatry. (1997) 38:581–6. 10.1111/j.1469-7610.1997.tb01545.x 9255702

[17] Goodman R. Psychometric properties of the strengths and difficulties questionnaire. J Am Acad Child Adolesc Psychiatry. (2001) 40:1337–45. 10.1097/00004583-200111000-00015 11699809

[18] Dorji T Totland Ø Moe SR Hopping KA Pan J Klein JA. Plant functional traits mediate reproductive phenology and success in response to experimental warming and snow addition in Tibet. Global Change Biol. (2013) 19:459–72. 10.1111/gcb.12059 23504784

[19] Popow C Ohmann S von Gontard A Paulus F. Computerspielabhängigkeit bei kindern und jugendlichen – ein überblick. Monatsschrift Kinderheilkunde. (2019) 167:124–30. 10.1007/s00112-018-0617-9

[20] Chan PA Rabinowitz T. A cross-sectional analysis of video games and attention deficit hyperactivity disorder symptoms in adolescents. Annal General Psychiatry. (2009) 5:16–27. 10.1186/1744-859X-5-16 17059614 PMC1635698

[21] Ko CH Yen JY Chen CS Yeh YC Yen CF. Predictive values of psychiatric symptoms for Internet addiction in adolescents: a 2-year prospective study. Archiv Pediatric Adolesc Med. (2009) 163:937–43. 10.1001/archpediatrics.2009.159 19805713

[22] Kietglaiwansiri T. Pattern of video game use in children with attention-deficit–hyperactivity disorder and typical development. Pediatrics Int. (2018) 60:523–8. 10.1111/ped.13564 29573063

[23] Koepp MJ Gunn RN Lawrence AD Cunningham VJ Dagher A Jones T. Evidence for striatal dopamine release during a video game. Nature. (1998) 393:266–8. 10.1038/30498 9607763

[24] Lorenz RC Gleich T Gallinat J Kühn S. Video game training and the reward system. Front Hum Neurosci. (2015) 9:1–9. 10.3389/fnhum.2015.00040 25698962 PMC4318496

[25] Gentile DA Hyekyung C Liau A Sim T Li D Fung D. Pathological video game use among youths: a 2-year longitudinal study. Pediatrics. (2011) 127:318–29. 10.1542/peds.2010-1353 21242221

[26] Lemmens JS Valkenburg PM Peter J. The effects of pathological gaming on aggressive behavior. J Youth Adolesc. (2011) 40:38–47. 10.1007/s10964-010-9558-x 20549320 PMC3003785

[27] Suissa A. Cyberaddictions: toward a psychosocial perspective. Addict Behav. (2014) 39:1914–8. 10.1016/j.addbeh.2014.07.027 25173593

[28] Yen JY Yen CF Chen CS Tang TC Ko CH. The association between adult ADHD symptoms and Internet addiction among college students: the gender difference. Cyberpsychol Behav. (2009) 12:187–91. 10.1089/cpb.2008.0113 19072077

[29] Bioulac S Arfi L Bouvard M. Attention deficit/ hyperactivity disorder and video games: a comparative study of hyperactive and control children. Eur Psychiatry. (2008) 23:134–4. 10.1016/j.eurpsy.2007.11.002 18206354

[30] Ra CK. Digital media and symptoms of attention-deficit/hyperactivity disorder in adolescents. J Am Med Assoc. (2018) 320:255–63. 10.1001/jama.2018.8931 30027248 PMC6553065

[31] Poulain T Vogel M Neef M Abicht F Hilbert A Genuneit J. Reciprocal associations between electronic media use and behavioral difficulties in preschoolers. Int J Environ Res Public Health. (2018) 15:814. 10.3390/ijerph15040814 29690498 PMC5923856

[32] Pagani LS Lévesque-Seck F Fitzpatrick C. Prospective associations between televiewing at toddlerhood and later self-reported social impairment at middle school in a Canadian longitudinal cohort born in 1997/1998. Psychol Med. (2016) 46:3329–37. 10.1017/S0033291716001689 27618949

[33] Livingstone S Smith PK. Annual research review: harms experienced by child users of online and mobile technologies: the nature, prevalence and management of sexual and aggressive risks in the digital age. J Child Psychol Psychiatry. (2014) 55:635–54. 10.1111/jcpp.12197 24438579

[34] Lin LY Cherng RJ Chen YJ Chen YJ Yang HM. Effects of television exposure on developmental skills among young children. Infant Behav Dev. (2015) 38:20–6. 10.1016/j.infbeh.2014.12.005 25544743

[35] Griffiths MD. Conceptual issues concerning internet addiction and internet gaming disorder. Int J Mental Health Addict. (2018) 16:233–39. 10.1007/s11469-017-9818-z 29491772 PMC5814513

[36] Canadian Paediatric Society. Screen time and young children: promoting health and development in a digital world. Paediatr Child Health. (2017) 22:461–8. 10.1093/pch/pxx123 29601064 PMC5823000

[37] Kwon JH Chung CS Lee J. The effects of escape from self and interpersonal relationship on the pathological use of Internet games. Community Mental Health J. (2011) 47:113–21. 10.1007/s10597-009-9236-1 19701792

[38] land TH Bjelland M Lien N Bergh IH Gebremariam MK Grydeland M. Adolescents prospective screen time by gender and parental education, the mediation of parental influences. Int J Behav Nutr Phys Act. (2013). 10:89. 23829607 10.1186/1479-5868-10-89 PMC3718651

[39] Radesky JS Christakis DA. Increased screen time: implications for early childhood development and behavior. Pediatric Clin. (2016) 63:827–39. 10.1016/j.pcl.2016.06.006 27565361

[40] Linebarger DL. Contextualizing video game play: the moderating effects of cumulative risk and parenting styles on the relations among video game exposure and problem behaviors. Psychol Popular Media Culture. (2015) 4:375. 10.1037/ppm0000069

[41] Minges KE Salmon J Dunstan DW Owen N Chao A Whittemore R. Reducing youth screen time: qualitative metasynthesis of findings on barriers and facilitators. Health Psychol. (2015) 34:381. 10.1037/hea0000172 25822054 PMC4456186

[42] Solomon-Moore E Toumpakari Z Sebire SJ Thompson JL Lawlor DA Jago R. Roles of mothers and fathers in supporting child physical activity: a cross-sectional mixed-methods study. BMJ Open. (2018) 8:19732. 10.1136/bmjopen-2017-019732 29358449 PMC5781024

[43] Schneider LA King DL Delfabbro PH. Family factors in adolescent problematic Internet gaming: a systematic review. J Behav Addict. (2017) 6:321–33. 10.1556/2006.6.2017.035 28762279 PMC5700711

[44] Solinski B. À la marge de la lecture et du ludique: les livres-jeux. Sciences du jeu. (2017) 7:777. 10.4000/sdj.777

[45] Granic I Lobel A Engels R. The benefits of playing video games. Am Psychol. (2014) 69:66–78. 10.1037/a0034857 24295515

